# Cognitive Frailty as a Predictor of Mortality in Older Adults: A Longitudinal Study in Peru

**DOI:** 10.3389/fmed.2022.910005

**Published:** 2022-06-22

**Authors:** Diego A. Vargas-Torres-Young, Leslie Salazar-Talla, Sofia Cuba-Ruiz, Diego Urrunaga-Pastor, Fernando M. Runzer-Colmenares, Jose F. Parodi

**Affiliations:** ^1^Universidad Científica del Sur, Facultad de Ciencias de la Salud, Carrera de Medicina Humana, Lima, Peru; ^2^Grupo Estudiantil de Investigación en Salud Mental (GISAM), Sociedad Científica de Estudiantes de Medicina de la Universidad de San Martin de Porres, Lima, Peru; ^3^Facultad de Medicina Humana, Universidad de San Martin de Porres, Lima, Peru; ^4^Universidad de San Martin de Porres, Facultad de Medicina Humana, Centro de Investigación del Envejecimiento (CIEN), Lima, Peru

**Keywords:** cognitive frailty, cognitive impairment, frailty, mortality, older adult, aging

## Abstract

**Objective:**

To evaluate the role of cognitive frailty and its components as risk factors of mortality in older adults of the *Centro Médico Naval* (CEMENA) in Callao, Peru during 2010-2015.

**Methods:**

We performed a secondary analysis of data from a prospective cohort that included older adults (60 years and older) treated at the CEMENA Geriatrics service between 2010–2015. Frailty was defined as the presence of three or more criteria of the modified Fried Phenotype. Cognitive impairment was assessed using the Peruvian version of the Mini Mental State Examination (MMSE), considering a score <21 as cognitive impairment. Cognitive frailty was defined as the coexistence of both. In addition, we included sociodemographic characteristics, medical and personal history, as well as the functional evaluation of each participant.

**Results:**

We included 1,390 older adults (mean follow-up: 2.2 years), with a mean age of 78.5 ± 8.6 years and 59.6% (*n* = 828) were male. Cognitive frailty was identified in 11.3% (*n* = 157) and 9.9% (*n* = 138) died during follow-up. We found that cognitive frailty in older adults (aHR = 3.57; 95%CI: 2.33–5.49), as well as its components, such as sedentary behavior and cognitive impairment (aHR = 7.05; 95%CI: 4.46–11.13), weakness and cognitive impairment (aHR = 6.99; 95%CI: 4.41–11.06), and exhaustion and cognitive impairment (aHR = 4.51; 95%CI: 3.11–6.54) were associated with a higher risk of mortality.

**Conclusion:**

Cognitive frailty and its components were associated with a higher risk of mortality in older adults. It is necessary to develop longitudinal studies with a longer follow-up and that allow evaluating the effect of interventions in this vulnerable group of patients to limit adverse health outcomes, including increased mortality.

## Introduction

During aging, the presence of multiple subclinical comorbidities and stressors can exacerbate the decrease in physiological reserves in various systems, causing homeostatic imbalance or frailty (1). Frailty results in the inability to perform basic activities of daily living (2), neurocognitive disorders (3) and an increased risk of mortality (4). In addition, frailty can increase the risk of future cognitive decline and vice versa (5–8). Cognitive impairment prevalence varies from 12.05 to 33.7% in frail older adults (9–11), with frailty being associated with poorer cognitive performance (12), and the coexistence of the two inducing a higher risk of adverse outcomes such as dementia, disability, hospitalizations, and death (13).

Coexistence of frailty and cognitive impairment is common and its prevalence in older adults varies from 10.3 to 42.8% (14–16), and therefore, a syndrome encompassing both (17) was defined as cognitive frailty in 2013. This syndrome excludes the presence of Alzheimer's disease and other dementias (18). Cognitive frailty refers to brain frailty that may be associated with neuropathological changes related to Alzheimer's disease or other neurodegenerative conditions (19). This is a potentially reversible clinical entity with an important goal of secondary prevention in the asymptomatic or early stage of dementia (20). Likewise, it predisposes older adults to more complex and serious outcomes (18), increasing the risk of dementia and all-cause mortality by approximately 4.01 and 3.4 fold, respectively (21, 22), being greater than the risk attributed to each syndrome separately (frailty and cognitive impairment increase in 1.8 and 1.3 mortality risk fold, respectively) (14).

Cognitive frailty as a risk factor for mortality has been described in systematic reviews (21, 23) and previous studies conducted in Asian countries (22, 24–26) and Europe (9), but the number of studies in in Latin American older adults is fewer (27, 28). Health systems in Latin America are fragmented and do not provide quality care to all population groups (29). In Peru, the situation is similar, with poverty limiting access to health services to older adults, who represent a vulnerable population due to the high prevalence of geriatric syndromes and the risk of adverse outcomes (30). It is important to identify early cognitive frailty because it is a reversible condition prior to dementia, so we could avoid adverse outcomes by acting promptly and it would be beneficial in the Peruvian context. For this reason, the objective of this study was to evaluate the role of cognitive frailty and its components as risk factors of mortality in older adults in Peru during the period from 2010 to 2015.

## Materials and Methods

### Study Design, Population, and Sample

We performed a secondary analysis of data from a prospective cohort that included 1891 older adults (60 years and over) enrolled in the Geriatrics Service of the *Centro Médico Naval* (CEMENA) “Cirujano Mayor Santiago Távara” during the period 2010–2015. The primary objective was to evaluate the prevalence and factors associated with frailty in older adults from CEMENA. In addition, other studies have been carried out with this database (31–34). The primary study included all the participants evaluated in the CEMENA Geriatrics Service from 2010 to 2015. For the secondary data analysis, we excluded participants with no record of the variables of interest.

Participants were enrolled in 2010 and followed annually until 2015. Likewise, a new group of older adults was enrolled annually and followed until 2015. We did not perform any additional measurement of baseline measurements, only mortality was assessed during follow-up. The mean follow-up was 2.2 years. Participants were chosen using non-probabilistic convenience sampling. A total of 1891 individuals were enrolled in the database and 501 were excluded for not having the variables of interest. Thus, 1390 older adults were finally analyzed. A statistical power of 100% was calculated for the final sample size based on a hazard ratio (HR) of 3.0 reported by Feng L. et al. (25).

### Variables

#### Outcome Variable: Mortality

Mortality was defined as death by all causes in the elderly registered by the CEMENA Epidemiological Surveillance Office during the follow-up period.

#### Exposure Variables

##### Frailty

We evaluated frailty using the modified Fried Phenotype, which consists of five criteria. (1) Exhaustion: defined using the following questions from the geriatric depression scale (35, 36): (a) Do you feel full of energy?; (b) Do you feel that you cannot go on?; (c) Do you feel that everything you do is an effort? Exhaustion was considered with two or more positive responses (37); (2) Weight loss: defined as a positive response to the following question taken from the Edmonton questionnaire (38): “Have you recently lost enough weight that your clothes are too loose?; (3) Weakness: defined as the recording of grip strength <16 kg in women and <27 kg in older men using a dynamometer (39); (4) Sedentary behavior: evaluated by the application of the Physical Activity Scale for the Elderly (PASE) and was considered positive with a score <64 in men and <52 in women (40, 41); and (5) Slow gait speed: defined as a walking speed <0.8 m/s or in cases in which the participant could not complete the four meter walk (39). The highest time recorded in each participant was considered. Frailty was defined as an older adult with three or more criteria.

##### Cognitive Impairment

We used the Peruvian version of the Mini Mental State Examination (MMSE), which is divided into five sections and has a maximum score of 30 points, with a higher score being interpreted as better cognitive performance. A score <21 points was considered as cognitive impairment (42).

##### Cognitive Frailty

Cognitive frailty is defined by the International Academy of Nutrition and Aging and the International Association of Gerontology and Geriatrics as the simultaneous presence of frailty (according to the phenotypic model) and cognitive impairment, excluding neurodegenerative causes or definite dementia (18). The term was coined in view of extensive evidence highlighting the association between these two geriatric syndromes.

#### Other Variables

##### Sociodemographic Characteristics

We collected the following sociodemographic characteristics: sex (male, female), age (60–70 years, 71–80 and ≥81), marital status (single, married/cohabiting, divorced/widowed), educational level ( ≤ 11 years or >11) and whether the participant lived alone (yes, no).

##### Medical and Personal History

We created a variable that included the following comorbidities: high blood pressure, type 2 diabetes mellitus, heart failure, chronic kidney disease, chronic obstructive pulmonary disease, arterial insufficiency, history of depression, urinary incontinence, and overweight or obesity according to the body mass index. In addition, by self-reporting we evaluated the history of tobacco consumption (no, yes) and alcohol consumption (no, yes), hospitalizations in the last year (no, yes), the number of prescribed medications and falls in the last year (no, yes). We obtained these variables from the participant's medical records.

##### Functional Evaluation

We evaluated functional dependence in basic activities of daily living (BADL) using the Barthel index, which evaluates 10 activities and has a maximum score of 100. We defined disability as a score <100 (43).

### Statistical Analysis

We used the statistical package STATA^®^ v17.0 (StataCorp, TX, USA) to perform the analysis. We did not have follow-up loss of the participants. Descriptive results corresponding to the qualitative variables are described using absolute and relative frequencies, while the quantitative variables are shown using measures of central tendency and dispersion. We performed the bivariate analysis using Pearson's chi-square test to compare the covariates of interest and the exposure variables (cognitive frailty, frailty, and cognitive impairment) and outcomes. In addition, we used the Student's *t*-test or the Mann-Whitney *U*-test to evaluate the differences between the numerical covariates and the exposure and outcome variables. We performed crude and adjusted Cox regression models to assess the association between cognitive frailty and all-cause mortality in the study sample. In addition, we evaluated the association between the components of cognitive frailty and the incidence of mortality in the study participants. The adjusted model included the following variables: sex, age, educational level, comorbidities, history of tobacco use, history of alcohol use, number of drugs prescribed, functional dependence for BADL and falls in the last year. We chose these variables using the classical confusion criteria and the description of their association in the literature (44–48). Crude (cHR) and adjusted (aHR) hazard ratios with their 95% confidence intervals (95%CI) were calculated. Likewise, a Kaplan-Meier curve was constructed to evaluate the survival of the participants according to the presence or absence of cognitive frailty and they were compared using the Log-rank test.

### Ethical Aspects

This secondary analysis was reviewed and approved by the institutional research ethics committee of the Universidad Científica del Sur, in Lima, Peru (151-2021-PREB15). Since this study involved analysis of secondary data, no additional measurement was performed in the participants. In addition, the primary study was approved by the CEMENA ethics committee, and the participants signed informed consent prior to entering the study.

## Results

### General Characteristics of the Sample and Bivariate Analysis According to the Exposure Variables

**Figure 1:**
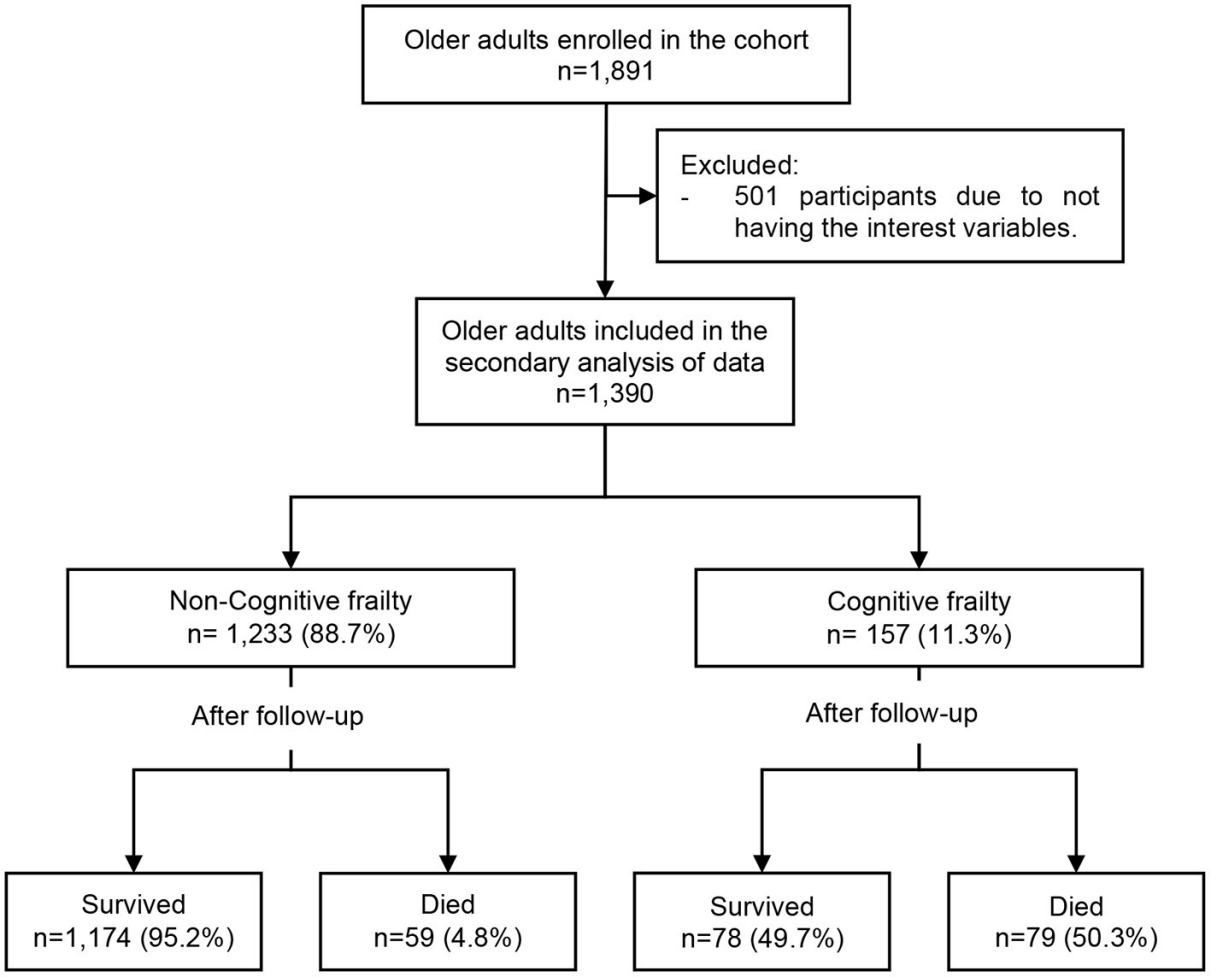
Flowchart of the sample selection.

The cohort study enrolled 1,891 older adults, and we excluded 501 due to not having the variables of interest for this secondary data analysis (Figure 1). Then, we analyzed 1,390 older adults with a mean age of 78.5 ± 8.6 years and 59.6% (*n* = 828) were male. In addition, 78.9% (*n* = 1,097) studied for more than 11 years, the median number of years of retirement was 21 (interquartile range [IQR]: 12–28), 84.6% (*n* = 1,176) did not live alone and 30.9% (*n* = 430) had 3 or more comorbidities. On the other hand, we found that 73.2% (*n* = 1,017) had a history of tobacco consumption, 61.6% (*n* = 856) had functional dependence for BADL, 51.1% (*n* = 711) had been hospitalized during the previous year and the median number of medications prescribed was 3 (IQR: 2–6). It was found that 11.3% (*n* = 157) of the participants had cognitive frailty, 24.0% (*n* = 333) were frail while 18.9% (*n* = 263) had cognitive impairment, and the incidence of mortality was 9.9% (*n* = 138). In addition, we found a greater percentage of male older adults (73.9 vs. 57.8%; *p* < 0.001), higher mean of age (80.4 vs. 78.2 years; *p* = 0.003), less median years of retirement (16 vs. 22; *p* = 0.030), and a higher median of drugs prescribed (8 vs. 3; *p* < 0.001) in cognitive frailty group compared with the non-exposed group (Table 1).

**Table 1:** Descriptive and bivariate analyses according to exposure variables (*n* = 1,390).

**Variables**	***n***	**%**	**Cognitive frailty**		***P* value**	**Frailty**		***P* value**	**Cognitive impairment**		***P* value**
			**No 88.7%**	**Yes 11.3%**		**No 76.0%**	**Yes 24.0%**		**No 81.1%**	**Yes 18.9%**	
			**(*n* = 1,233)**	**(*n* = 157)**		**(*n* = 1,057)**	**(*n* = 333)**		**(*n* = 1,127)**	**(*n* = 263)**	
**Sex**					<0.001			<0.001			0.457
Female	562	40.4	521 (42.2)	41 (26.1)		473 (44.8)	89 (26.7)		461 (40.9)	101 (38.4)	
Male	828	59.6	712 (57.8)	116 (73.9)		584 (55.3)	244 (73.3)		666 (59.1)	162 (61.6)	
**Age**	78.5 ± 8.6		78.2 ± 8.5	80.4 ± 8.8	0.003	77.3 ± 8.1	82.1 ± 9.0	<0.001	78.2 ± 8.6	79.7 ± 8.4	0.008
60–70 years old	221	15.9	205 (16.6)	16 (10.2)	0.074	193 (18.3)	28 (8.4)	<0.001	193 (17.1)	28 (10.6)	0.016
71–80 years old	623	44.8	553 (44.9)	70 (44.6)		497 (47.0)	126 (37.8)		506 (44.9)	117 (44.5)	
≥81 years old	546	39.3	475 (38.5)	71 (45.2)		367 (34.7)	179 (53.8)		428 (38.0)	118 (44.9)	
**Marital status**					0.377			0.863			0.115
Single	39	2.8	37 (3.0)	2 (1.3)		31 (2.9)	8 (2.4)		36 (3.2)	3 (1.1)	
Married/Cohabitating	1,098	79.0	975 (79.1)	123 (78.3)		835 (79.0)	263 (79.0)		881 (78.2)	217 (82.5)	
Divorced/Widower	253	18.2	221 (17.9)	32 (20.4)		191 (18.1)	62 (18.6)		210 (18.6)	43 (16.4)	
**Educational level**					0.984			0.048			0.271
≤ 11 years	293	21.1	260 (21.1)	33 (21.0)		210 (19.9)	83 (24.9)		231 (20.5)	62 (23.6)	
>11 years	1,097	78.9	973 (78.9)	124 (79.0)		847 (80.1)	250 (75.1)		896 (79.5)	201 (76.4)	
**Military rank**					0.104			0.121			0.003
Subaltern	721	51.9	638 (51.7)	83 (52.9)		533 (50.4)	188 (56.5)		581 (51.6)	140 (53.2)	
Officer	135	9.7	127 (10.3)	8 (5.1)		109 (10.3)	26 (7.8)		124 (11.0)	11 (4.2)	
Civilian	534	38.4	468 (38.0)	66 (42.0)		415 (39.3)	119 (35.7)		422 (37.4)	112 (42.6)	
**Years of retirement**	21	(122–8)	22 (13–28)	16 (10–28)	0.030	21 (14–28)	19 (92–9)	0.528	22 (13–28)	17 (92–8)	0.036
**Living alone**					0.327			0.359			0.923
No	1,176	84.6	1,039 (84.3)	137 (87.3)		889 (84.1)	287 (86.2)		954 (84.7)	222 (84.4)	
Yes	214	15.4	194 (15.7)	20 (12.7)		168 (15.9)	46 (13.8)		173 (15.3)	41 (15.6)	
**Comorbidities**	2	(1–3)	2 (1–3)	2 (1–2)	0.165	2 (1–3)	2 (1–3)	0.002	2 (1–3)	2 (13–)	0.068
0	121	8.7	111 (9.0)	10 (6.4)	0.056	89 (8.4)	32 (9.6)	0.020	102 (9.1)	19 (7.2)	0.038
1	400	28.8	344 (27.9)	56 (35.7)		285 (27.0)	115 (34.5)		307 (27.2)	93 (35.4)	
2	439	31.6	385 (31.2)	54 (34.4)		338 (32.0)	101 (30.3)		356 (31.6)	83 (31.6)	
≥3	430	30.9	393 (31.9)	37 (23.6)		345 (32.6)	85 (25.5)		362 (32.1)	68 (25.8)	
**BMI** ^**a**^	26.1 ± 5.7		26.2 ± 5.6	25.3 ± 6.3	0.094	26.2 ± 5.6	25.7 ± 5.8	0.204	26.4 ± 5.5	24.7 ± 6.1	<0.001
**History of tobacco consumption**					0.352			0.005			0.145
No	373	26.8	326 (26.4)	47 (29.9)		264 (25.0)	109 (32.7)		293 (26.0)	80 (30.4)	
Yes	1,017	73.2	907 (73.6)	110 (70.1)		793 (75.0)	224 (67.3)		834 (74.0)	183 (69.6)	
**History of alcohol consumption**					0.511			0.298			0.023
No	769	55.3	686 (55.6)	83 (52.9)		593 (56.1)	176 (52.9)		640 (56.8)	129 (49.1)	
Yes	621	44.7	547 (44.4)	74 (47.1)		464 (43.9)	157 (47.1)		487 (43.2)	134 (50.9)	
**Functional dependance in BADL** ^**b**^					0.203			0.444			0.401
No	534	38.4	481 (39.0)	53 (33.8)		412 (39.0)	122 (36.6)		427 (37.9)	107 (40.7)	
Yes	856	61.6	752 (61.0)	104 (66.2)		645 (61.0)	211 (63.4)		700 (62.1)	156 (59.3)	
**Hospitalizations in the last year**					0.070			0.001			0.729
No	679	48.9	613 (49.7)	66 (42.0)		544 (51.5)	135 (40.5)		548 (48.6)	131 (49.8)	
Yes	711	51.1	620 (50.3)	91 (58.0)		513 (48.5)	198 (59.5)		579 (51.4)	132 (50.2)	
**Number of drugs prescribed**	3 (2–6)		3 (2–4)	8 (7–9)	<0.001	3 (2–4)	6 (3–8)	<0.001	3 (2–4)	8 (6–8)	<0.001
**Exhaustion**					<0.001			<0.001			<0.001
No	1,075	77.3	1,021 (82.8)	54 (34.4)		916 (86.7)	159 (47.8)		939 (83.3)	136 (51.7)	
Yes	315	22.7	212 (17.2)	103 (65.6)		141 (13.3)	174 (52.2)		188 (16.7)	127 (48.3)	
**Weight loss**					<0.001			<0.001			0.020
No	915	65.8	840 (68.1)	75 (47.8)		795 (75.2)	120 (36.0)		758 (67.3)	157 (59.7)	
Yes	475	34.2	393 (31.9)	82 (52.2)		262 (24.8)	213 (64.0)		369 (32.7)	106 (40.3)	
**Weakness**					<0.001			<0.001			<0.001
No	945	68.0	925 (75.0)	20 (12.7)		872 (82.5)	73 (21.9)		848 (75.2)	97 (36.9)	
Yes	445	32.0	308 (25.0)	137 (87.3)		185 (17.5)	260 (78.1)		279 (24.8)	166 (63.1)	
**Sedentary behavior**					<0.001			<0.001			<0.001
No	762	54.8	762 (61.8)	0 (0)		717 (67.8)	45 (13.5)		710 (63.0)	52 (19.8)	
Yes	628	45.2	471 (38.2)	157 (100)		340 (32.2)	288 (86.5)		417 (37.0)	211 (80.2)	
**Slow gait speed**					<0.001			<0.001			<0.001
No	954	68.6	908 (73.6)	46 (29.3)		855 (80.9)	99 (29.7)		834 (74.0)	120 (45.6)	
Yes	436	31.4	325 (26.4)	111 (70.7)		202 (19.1)	234 (70.3)		293 (26.0)	143 (54.4)	
**Falls in the last year**					0.094			<0.001			0.888
No	555	39.9	502 (40.7)	53 (33.8)		455 (43.1)	100 (30.0)		451 (40.0)	104 (39.5)	
Yes	835	60.1	731 (59.3)	104 (66.2)		602 (56.9)	233 (70.0)		676 (60.0)	159 (60.5)	
**Mortality**					<0.001			<0.001			<0.001
No	1,252	90.1	1,174 (95.2)	78 (49.7)		1,009 (95.5)	243 (73.0)		1,108 (98.3)	144 (54.8)	
Yes	138	9.9	59 (4.8)	79 (50.3)		48 (4.5)	90 (27.0)		19 (1.7)	119 (45.2)	

^a^*Body mass index; ^b^Basic activities of daily life*.

### Bivariate Analysis According to Mortality in the Study Sample

The group with cognitive frailty presented a higher incidence of mortality compared to those without this condition (50.3 vs. 4.8%; *p* < 0.001). In addition, mortality was higher in frail participants (27.0 vs. 4.5%; *p* < 0.001) or those with cognitive impairment (45.2 vs. 1.7%; *p* < 0.001) compared to individuals without these conditions. However, there were no statistically significant differences in relation to mortality and sex, marital status, educational level, military rank, years of retirement, living alone, comorbidities, body mass index, history of consumption of alcohol, hospitalizations in the last year and self-reported weight loss (Table 2).

**Table 2:** Descriptive and bivariate analyses of the study variables based on all-cause mortality (*n* = 1,390).

**Variables**	**Mortality**		***P* value**
	**No 90.1%**	**Yes 9.9%**	
	**(*n* = 1,252)**	**(*n* = 138)**	
**Cognitive frailty**			<0.001
No	1,174 (95.2)	59 (4.8)	
Yes	78 (49.7)	79 (50.3)	
**Frailty**			<0.001
No	1,009 (95.5)	48 (4.5)	
Yes	243 (73.0)	90 (27.0)	
**Cognitive impairment**			<0.001
No	1,108 (98.3)	19 (1.7)	
Yes	144 (54.8)	119 (45.2)	
**Sex**			0.214
Female	513 (91.3)	49 (8.7)	
Male	739 (89.3)	89 (10.7)	
**Age**	78.3 ± 8.6	80.2 ± 8.4	0.012
60–70 years old	206 (93.2)	15 (6.8)	0.078
71–80 years old	565 (90.7)	58 (9.3)	
≥81 years old	481 (88.1)	65 (11.9)	
**Marital status**			0.138
Single	36 (92.3)	3 (7.7)	
Married/Cohabitating	980 (89.2)	118 (10.8)	
Divorced/Widower	236 (93.3)	17 (6.7)	
**Educational level**			0.119
≤ 11 years	271 (92.5)	22 (7.5)	
>11 years	981 (89.4)	116 (10.6)	
**Military rank**			0.126
Subaltern	649 (90.0)	72 (10.0)	
Officer	128 (94.8)	7 (5.2)	
Civilian	475 (89.0)	59 (11.0)	
**Years of retirement**	21 (13–28)	19 (10–29)	0.825
**Living alone**			0.153
No	1,065 (90.6)	111 (9.4)	
Yes	187 (87.4)	27 (12.6)	
**Comorbidities**	2 (1–3)	2 (1–2)	0.064
0	111 (91.7)	10 (8.3)	0.066
1	350 (87.5)	50 (12.5)	
2	392 (89.3)	47 (10.7)	
≥3	399 (92.8)	31 (7.2)	
**BMI** ^**a**^	26.2 ± 5.6	25.2 ± 6.7	0.054
**History of tobacco consumption**			0.042
No	346 (92.8)	27 (7.2)	
Yes	906 (89.1)	111 (10.9)	
**History of alcohol consumption**			0.510
No	689 (89.6)	80 (10.4)	
Yes	563 (90.7)	58 (9.3)	
**Functional dependance in BADL** ^**b**^			<0.001
No	444 (83.2)	90 (16.8)	
Yes	808 (94.4)	48 (5.6)	
**Hospitalizations in the last year**			0.941
No	612 (90.1)	67 (9.9)	
Yes	640 (90.0)	71 (10.0)	
**Number of drugs prescribed**	3 (2–4)	8 (7–9)	<0.001
**Exhaustion**			<0.001
No	1,017 (94.6)	58 (5.4)	
Yes	235 (74.6)	80 (25.4)	
**Weight loss**			0.269
No	830 (90.7)	85 (9.3)	
Yes	422 (88.8)	53 (11.2)	
**Weakness**			<0.001
No	900 (95.2)	45 (4.8)	
Yes	352 (79.1)	93 (20.9)	
**Sedentary behavior**			<0.001
No	735 (96.5)	27 (3.5)	
Yes	517 (82.3)	111 (17.7)	
**Slow gait speed**			<0.001
No	892 (93.5)	62 (6.5)	
Yes	360 (82.6)	76 (17.4)	
**Falls in the last year**			0.010
No	514 (92.6)	41 (7.4)	
Yes	738 (88.4)	97 (11.6)	

^a^*Body mass index; ^b^Basic activities of daily life*.

### Cognitive Frailty as a Risk Factor for Mortality in Older Adults

**Figure 2:**
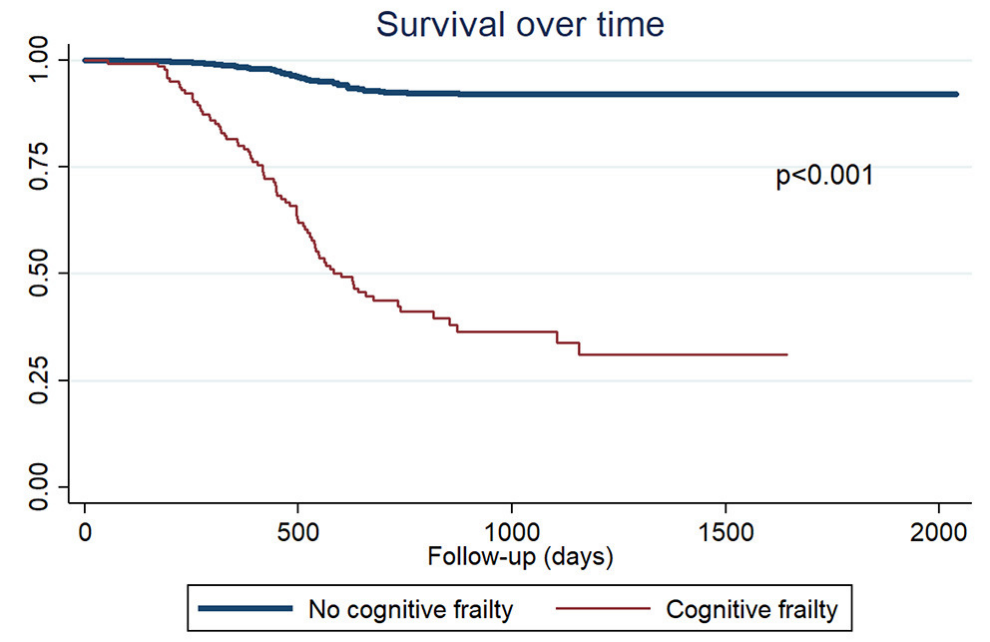
Kaplan-Meier survival curves according to the cognitive frailty phenotype.

The adjusted Cox regression analysis showed that cognitive frailty (aHR = 3.57; 95%CI: 2.33–5.49; *p* < 0.001) was a risk factor for mortality in older adults. In addition, we evaluated the association of the components of cognitive frailty, finding that exhaustion and cognitive impairment (aHR = 4.51; 95%CI: 3.11–6.54; *p* < 0.001), weight loss and cognitive impairment (aHR = 1.68; 95%CI: 1.06–2.67; *p* = 0.027), weakness and cognitive impairment (aHR = 6.99; 95%CI: 4.41–11.06; *p* < 0.001), sedentary behavior and cognitive impairment (aHR = 7.05; 95%CI: 4.46–11.13; *p* < 0.001), slow gait speed and cognitive impairment (aHR = 2.61; 95%CI: 1.76–3.85; *p* < 0.001) were associated with a higher risk of mortality (Table 3). In addition, the group of patients with cognitive frailty presented a lower survival (*p* < 0.001) (Figure 2). The mean survival of cognitive frailty group was 1.6 years (median: 1.4), while in the non-exposed group the mean survival was 2.3 years (median: 1.7).

**Table 3:** Cox regression models to evaluate the association between the cognitive frailty phenotype and the risk of mortality in the study sample.

	**Crude**			**Adjusted**		
**Exposure variables**	**cHR**	**95%CI**	***P* value**	**aHR^**a**^**	**95%CI**	***P* value**
**Cognitive frailty**						
No	Reference	–	–	Reference	–	–
Yes	12.61	8.98–17.71	<0.001	3.57	2.33–5.49	<0.001
*Cognitive frailty phenotype components*						
**Exhaustion** **+** **cognitive impairment**						
No	Reference	–	–	Reference	–	–
Yes	12.54	8.95–17.57	<0.001	4.51	3.11–6.54	<0.001
**Weight loss** **+** **cognitive impairment**						
No	Reference	–	–	Reference	–	–
Yes	5.80	4.00–8.41	<0.001	1.68	1.06–2.67	0.027
**Weakness** **+** **cognitive impairment**						
No	Reference	–	–	Reference	–	–
Yes	14.23	10.09–20.06	<0.001	6.99	4.41–11.06	<0.001
**Sedentary behavior** **+** **cognitive impairment**						
No	Reference		–	Reference	–	–
Yes	19.58	13.35–28.71	<0.001	7.05	4.46–11.13	<0.001
**Slow gait speed** **+** **cognitive impairment**						
No	Reference	–	–	Reference	–	–
Yes	10.74	7.67–15.04	<0.001	2.61	1.76–3.85	<0.001

^a^*Adjusted for: sex, age, educational level, comorbidities, history of tobacco consumption, history of alcohol consumption, number of drugs prescribed, functional dependence in BADL and falls in the last year*.

*aHR: adjusted hazard ratio; cHR: crude hazard ratio; CI: confidence interval*.

## Discussion

The present study evaluated 1,390 older adults, among whom two out of 10 were frail, two out of 10 had cognitive impairment, and one out of 10 had cognitive frailty. In addition, the latter was associated with a 3.57-fold increase in the risk of mortality. When evaluating the components of cognitive frailty, we found a higher incidence of mortality in older adults with a sedentary behavior, weakness, and exhaustion. Likewise, six out of 10 had disability in BADL or at least one fall in the last year and nine out of 10 had at least one comorbidity.

Our findings indicate the need for timely identification of cognitive frailty in primary care in order to reduce adverse outcomes. This is very important in our country due to the high prevalence of frailty (17.5 to 23.3%) and cognitive impairment (18.2 to 36.67%) described in several studies (49–54).

The prevalence of frailty, cognitive impairment, and cognitive frailty in the present study was 24, 18.9, and 11.3%, respectively. These prevalences are lower than those reported in previous studies in China, although the frequency of cognitive impairment was higher in one of these studies (48, 55). Likewise, a South Korean study reported a higher prevalence of cognitive impairment, but a lower frequency of frailty (14). On the other hand, a systematic review found a prevalence of cognitive frailty ranging from 2.5 to 50% in cohort studies using different operational definitions (23).

We found that cognitive frailty was associated with an increased risk of mortality in older Peruvian adults. This finding is similar to what has been described in China (48, 55), South Korea (14) and France (9). However, these studies were heterogeneous in relation to follow-up time, sample size, age of the older adults, and the instruments used to measure frailty and cognitive impairment. Mortality risk assessment according to each component of cognitive frailty was not reported by any of the previous studies. Likewise, only one previous study used the modified version of the Fried phenotype (14). It should be noted that few studies has evaluated this association of interest in Latin American countries (27, 28, 44). One study evaluated the association of interest in older Mexican adults residing in the United States, using pre-frailty instead of frailty for the definition of cognitive frailty (44). Two previous studies conducted in older adults from Brazil evaluated the role of cognitive frailty as a predictor of mortality. One of them estimated the incidence of mortality, disability and falls (28) after 1 year of follow-up, while the other only evaluated mortality, but after 10 years (27). Both evaluated frailty by accumulation of deficits (one using the FRAIL questionnaire and the other using the Frailty Index), while we used the Fried phenotype. Both quantified mortality risk not only for older adults with cognitive frailty, but also for prefrail participants with cognitive impairment, however, we explored each component of cognitive frailty. The identification of accessible markers that, added to frailty, could increase the risk of mortality in older adults could be useful in Peru, whose health system is fragmented and does not allow rapid access to appointments, medications, or periodic control (56).

Several studies have described a lower degree of physical activity in older adults with a decrease in brain mass (57, 58). Likewise, a reduction of muscle strength and physical performance has been associated with cognitive deterioration (59). On the other hand, both syndromes share multiple risk factors such as advanced age itself (60), cardiovascular disease, mental disorders and lifestyles (61–63).

The presence of an inflammatory state mediated by cytokines in aging has been identified as an etiological factor in cognitive decline. It is known that increased concentrations of interleukins, specifically IL-6, favor memory decline. In addition, some infectious or proinflammatory processes, such as cancer, which are more frequent in the elderly, can lead to an increase in interleukins and subsequent inflammatory processes that can degrade cognitive capacity in the long term (64–66). Furthermore, chronic inflammation has been associated with poor physical performance and decreased muscle mass, as part of immunosenescence or inflammaging (7). In addition, previous studies have described cancer as a risk factor for frailty (67), both increasing the occurrence of adverse outcomes (68).

Reduced testosterone and other androgen hormones may be implicated in the development of frailty and cognitive impairment. Testosterone could have a protective effect on cognition due to its role in promoting hippocampal synaptic plasticity and amyloid beta protein regulation (69). In addition, the decrease in testosterone levels due to aging is associated with a decrease in muscle mass, and therefore, with frailty (70). In addition, the role of insulin resistance has been described as a possible risk factor for the development of both conditions (71).

Other factors related to the development of cognitive frailty are vascular damage (72), vitamin D deficiency (73) and malnutrition (74). Nutrition is linked to cognitive impairment and frailty through sarcopenia, and oxidative stress may have an important role. Previous studies have described that Mediterranean diet (a diet high in antioxidants), was associated with less frailty and cognitive impairment (75, 76). In addition, nutrition could also be associated with frailty due to changes in behavior produced by cognitive impairment, which would affect the ability to eat (or remember to) or to accomplish a healthy eating plan (77). These pathophysiological pathways that respond to the multifactorial origin of cognitive frailty may be related to the increased risk of mortality evidenced in our study. This is due to a higher prevalence of comorbidities, less ability to maintain healthy lifestyles due to cognitive impairment and the consequent detriment to physical performance (78, 79).

Although the global prevalence of frailty varies significantly depending on the operational definition used and the characteristics of the population studied (4–59%) (80), we can affirm that it is a relevant syndrome among older adults. In Latin America, it is estimated that one in five elderly people is frail (81), with a prevalence of frailty of 24% in our study of the Peruvian population, thereby demonstrating the need to adapt health services to a population with greater demands.

While the prevalence of cognitive frailty is low and variable, ranging from 1 to 5% due to operational difficulties (60), it has been consistently identified as a risk factor for disability, morbidity and mortality in the elderly. However, due to its potential reversibility, it has been considered a possible intervention target to improve the quality of life in this population group.

Interventions aimed at addressing cognitive frailty include the promotion of exercise, a healthy diet, smoking cessation, psychological sessions, improvement of the social environment, and the control of variables such as weight, cholesterol, diabetes mellitus, and blood pressure (82–84). Interventions applied at various stages (pre-frailty, frailty and cognitive frailty) can help delay the development of frailty and improve the patient's adaptation to the physiological decrease in reserves (5, 7, 40, 82, 83, 85).

Although there is no consensus as to the best method for detecting cognitive frailty (86), our study found that two practical instruments available in daily clinical practice, such as the modified Fried phenotype and the MMSE, can predict a 3-fold higher risk of a mortality risk in patients with compared to those without cognitive frailty. This is especially useful in the context of the first level of care in countries with few available resources, such as Latin America.

Finally, this is the first study to describe an association between cognitive impairment and each component alone of the modified Fried phenotype and a higher risk of mortality (from 1.68 to 7.05 times higher depending on the component). This can have relevant implications due to the underdiagnosis of pre-frail states, in which only one or two criteria are present. Our findings highlight the importance of intervention in this group of patients to limit adverse health outcomes, including increased mortality.

This study has limitations: (1) The patients included did not have the same follow-up time, which could affect the incidence of mortality; (2) We included only older adults treated at CEMENA, which provides medical care to retired seafarers and their families, and thus, our findings may not be representative of the general population; (3) We did not exclude older adults with dementia in primary study data collection, because we did not evaluate them using the DSM-5 (gold standard); (4) We were unable to collect variables related to the type of medication received and the state of control of chronic diseases, which could affect the incidence of mortality; and (5) We did not collect the history of cancer as a variable, which could increase the risk of frailty and cognitive impairment in the study sample. Despite these limitations, our study is one of the first cohorts in Latin America which allowed evaluation of the role of cognitive frailty and its components as predictors of mortality in older adults. Our results allow us to identify cognitive frailty and its components as useful and practical markers in the first levels of healthcare. In the Peruvian context, these findings could be very important because they would allow the identification of risk groups in whom healthcare should be prioritized to avoid adverse outcomes. This would avoid the increase in the burden of the health system and would allow better care for older Peruvian adults.

In conclusion, our study found that cognitive frailty and its components are risk factors for mortality in older adults. Cognitive impairment associated with each component of the modified Fried phenotype was independently associated with an increased risk of mortality, with sedentary behavior, weakness, and exhaustion being of note. It is necessary to develop longitudinal studies with a longer follow-up time that allow evaluating the effect of interventions in this vulnerable group of patients to limit adverse health outcomes, including increased mortality.

## Data Availability Statement

The raw data supporting the conclusions of this article will be made available by the authors, without undue reservation.

## Ethics Statement

The studies involving human participants were reviewed and approved by the Institutional Research Ethics Committee of the Universidad Científica del Sur, in Lima, Peru (151-2021-PREB15). The patients/participants provided their written informed consent to participate in this study.

## Author Contributions

DV-T-Y, LS-T, SC-R, DU-P, FR-C, and JP participated in concept design and supervising the study. DU-P and FR-C conducted the statistical analysis. All the authors participated in manuscript writing, editing, final revision, and have read and agreed on the submitted manuscript, and also participated in this research and contributed to the final version of the manuscript. All authors contributed to the article and approved the submitted version.

## Conflict of Interest

The authors declare that the research was conducted in the absence of any commercial or financial relationships that could be construed as a potential conflict of interest.

## Publisher's Note

All claims expressed in this article are solely those of the authors and do not necessarily represent those of their affiliated organizations, or those of the publisher, the editors and the reviewers. Any product that may be evaluated in this article, or claim that may be made by its manufacturer, is not guaranteed or endorsed by the publisher.
